# The Chick Embryo Xenograft Model for Malignant Pleural Mesothelioma: A Cost and Time Efficient 3Rs Model for Drug Target Evaluation

**DOI:** 10.3390/cancers14235836

**Published:** 2022-11-26

**Authors:** Sarah E. Barnett, Anne Herrmann, Liam Shaw, Elisabeth N. Gash, Harish Poptani, Joseph J. Sacco, Judy M. Coulson

**Affiliations:** 1 Molecular Physiology & Cell Signalling, Institute of Systems, Molecular & Integrative Biology, University of Liverpool, Liverpool L69 3BX, UK; 2 Technology, Infrastructure & Environment Directorate, Faculty of Health & Life Sciences, University of Liverpool, Liverpool L69 7ZX, UK; 3 Molecular and Clinical Cancer Medicine, Institute of Systems, Molecular & Integrative Biology, University of Liverpool, Liverpool L69 7ZX, UK

**Keywords:** mesothelioma, chick embryo, CAM, xenograft, bioluminescence, fluorescence, histology, MRI, preclinical, 3Rs

## Abstract

**Simple Summary:**

Malignant pleural mesothelioma is a cancer of the lung lining, normally caused by asbestos, that develops decades after exposure. Chemotherapy, and recently more targeted drugs, show some benefit although only a minority of patients respond and invariably the cancer eventually escapes control. Several key genetic changes in mesothelioma differ from patient to patient, which may influence how their cancer responds to treatments. We have developed a new preclinical model using fertilised hen’s eggs as an alternative to laboratory rodents. Mesothelioma cells are labelled to allow monitoring of tumour growth and/or regression using fluorescence and longitudinal bioluminescence imaging in addition to histology. All cell lines tested efficiently form tumour nodules within seven days, supported by a blood supply and stromal chick cells. The model is rapid, cost effective, scalable, and adaptable with multiple potential endpoints, to enable evaluation of drug targets against the range of common mesothelioma genetic backgrounds.

**Abstract:**

Malignant pleural mesothelioma (MPM) has limited treatment options and poor prognosis. Frequent inactivation of the tumour suppressors *BAP1*, *NF2* and *P16* may differentially sensitise tumours to treatments. We have established chick chorioallantoic membrane (CAM) xenograft models of low-passage MPM cell lines and protocols for evaluating drug responses. Ten cell lines, representing the spectrum of histological subtypes and tumour suppressor status, were dual labelled for fluorescence/bioluminescence imaging and implanted on the CAM at E7. Bioluminescence was used to assess viability of primary tumours, which were excised at E14 for immunohistological staining or real-time PCR. All MPM cell lines engrafted efficiently forming vascularised nodules, however their size, morphology and interaction with chick cells varied. MPM phenotypes including local invasion, fibroblast recruitment, tumour angiogenesis and vascular remodelling were evident. Bioluminescence imaging could be used to reliably estimate tumour burden pre- and post-treatment, correlating with tumour weight and Ki-67 staining. In conclusion, MPM-CAM models recapitulate important features of the disease and are suitable to assess drug targets using a broad range of MPM cell lines that allow histological or genetic stratification. They are amenable to multi-modal imaging, potentially offering a time and cost-efficient, 3Rs-compliant alternative to rodent xenograft models to prioritise candidate compounds from in vitro studies.

## 1. Introduction

Malignant pleural mesothelioma (MPM) is an aggressive cancer of the lung lining, mainly caused by environmental exposure to asbestos. Despite strict asbestos regulation in most high-income countries, the global incidence of MPM has not yet peaked, and it is predicted to remain a significant cause of morbidity and mortality for decades [1]. MPM is commonly categorised as three main histopathological types, epithelioid, sarcomatoid or biphasic, which may represent a continuous spectrum of disease [2]. Invasion into the surrounding stroma is a diagnostic criterion [3]. MPM has extensive interactions with immune and stromal cells in the tumour microenvironment, in particular fibroblasts and endothelial cells. Asbestos causes fibrotic disease, with a desmoplastic reaction often evident in MPM that suggests involvement of cancer-associated fibroblasts (CAFs) [4]. Indeed, MPM recruits and activates CAFs to promote tumour progression through growth factor and cytokine networks in murine models [5]. Tumour angiogenesis also plays an important role in the pathogenesis of MPM, which commonly express vascular endothelial growth factor (VEGF) [6].

MPM is almost invariably incurable, even in early presentation, and chemotherapy benefits are very modest. Combination cisplatin and pemetrexed was the mainstay of treatment for over 20 years [7], with potential additional benefit from the VEGF antibody bevacizumab [8], until the recent licencing of immunotherapy. Combined nivolumab and ipilimumab treatment provides real clinical benefit [9], however not all patients are eligible, and most who are treated progress within a year. Therefore, an urgent need for further treatment options remains. Other targeted therapies have been slow to emerge for MPM, which lacks common oncogenic drivers, and instead is characterised by loss-of-function mutations in tumour suppressors, most commonly *BAP1*, *P16* and *NF2* [10,11]. Nevertheless, genetic loss of tumour suppressors or other epigenetic alterations may sensitise MPM to targeted therapies, with arginine deprivation and CDK4/6, PARP or EZH2 inhibitors now showing promise in early phase trials [12,13,14,15]. However, benefits vary widely with histological and genetic features in this heterogeneous disease and may be restricted to a small percentage of patients, whilst robust predictive biomarkers remain elusive. As more complete understanding of MPM biology emerges this offers hope for new therapeutic strategies and suitable patient stratification. However, to realise this potential, effective preclinical models are required to translate discovery science into clinical application.

Preclinical models that are currently used or in development for MPM were recently reviewed [16,17]. More than 100 cell lines derived from human MPM are available for in vitro culture to investigate cell biology or drug sensitivity [16]. Their inherent drawbacks, such as adaptation during two-dimensional (2D) culture, and a lack of heterogeneity, 3D architecture or microenvironment interactions, may be partially mitigated by using a sufficient number and diversity of low passage cell lines, growth as spheroid cultures [18], or co-culture with immune or stromal cells [19,20]. Organoid or explant models that better recapitulate tumour heterogeneity, 3D structure, and microenvironment in vitro require access to fresh MPM patient tissue. Explant cultures have been used successfully in MPM [21,22] although explants typically retain tumour architecture for just a few days and are prone to necrosis [23]. In contrast, proposed organoid cultures for MPM [16] could be sustained in longer term culture but lack contextual architecture. Currently, a vascularised 3D architecture and microenvironment can only be achieved using in vivo models in protected animals. Established human MPM cell lines or patient-derived tissues may be used for sub-cutaneous or orthotopic xenografts in immune compromised mice, although this typically precludes study of tumour/immune cell interactions [24,25,26]. Alternative immunocompetent in vivo MPM models rely on induction by asbestos or long carbon nanotubes in rodents, which can accelerate MPM development in genetically engineered mice with mutation of relevant tumour suppressor genes [27,28,29,30,31,32]. However, in addition to being expensive and slow to establish, these models often rely on procedures of high severity, and their utility may be limited by variable histopathology and species-specific gene associations [16]. Thus, a spectrum of MPM preclinical models is required for prioritising new therapeutic candidates and modelling different aspects of the human disease, to deliver meaningful preclinical data whilst supporting the replacement, reduction, and refinement of the use of animals (3Rs). Notably, a 3Rs compliant in vivo model that recapitulates aspects of 3D tumour architecture and microenvironment is currently lacking for MPM.

Fertilised hen’s eggs provide a non-protected in vivo model that does not require an animal licence until a specified stage of embryonic development, as defined by national regulations; in the UK this is two thirds of the gestation period. The chick embryo chorioallantoic membrane (CAM) is a rich source of oxygen and growth factors [33,34] and was first used to grow chicken sarcoma cells over a century ago [35]. A wide range of human cancer cells including melanoma, neuroblastoma, breast, colorectal and pancreatic cancer have subsequently been xenografted on the CAM [33]. However, despite one study providing proof of principle for use of patient-derived mesothelioma tissue [36], this has not been adopted by the field, and no CAM models have yet been developed with MPM cell lines. For other cancers, 3D vascularised tumours form within days, providing a rapid and cost-effective alternative to rodent xenografts. The chick embryo model allows monitoring of the major hallmarks of cancer including proliferation, angiogenesis, invasion, and metastasis, whilst recent studies demonstrate the feasibility of drug administration and utility of preclinical imaging [33,34,37,38,39,40]. We propose that incorporating such methodology into new MPM-CAM models will enhance the drug testing pipeline, for example by providing initial in vivo evaluation to reduce the use of protected animal models. We set out to develop robust standard operating protocols (SOPs) for establishing MPM-CAM xenograft models and to characterise the biology of the tumour nodules, which could in future enable a stratified approach to drug assessment.

Accordingly, we report here validated protocols that allow the efficient generation of xenografts on the CAM using a panel of well-characterised low passage MPM cell lines, which represent the spectrum of histopathology and tumour suppressor inactivation seen in the human disease. Importantly, the engrafted MPM nodules exhibit interactions with chick fibroblasts and vasculature, mimicking key facets of clinical MPM that are absent from in vitro cultures and which may influence therapeutic outcomes. We also show that bioluminescence imaging (BLI) can readily be used to evaluate MPM tumour burden over time, reducing inter-egg variability in tumour nodule measurements to minimise the number of embryos required to power studies. The protocols we describe for this new MPM-CAM preclinical model make it amenable to rapid, medium throughput evaluation of MPM cell biology in relation to genetics or therapeutics. Readouts, including multimodal imaging, transcriptional and histopathological analysis can be combined to monitor tumour size, survival, vascularisation, invasion, stromal composition, and proliferative capacity, and may in future be expanded to include markers of drug sensitivity or target engagement.

## 2. Materials and Methods

### 2.1. Cell Lines and Cell Culture

The MSTO-211H mesothelioma cell line [41] was obtained from ATCC. The other mesothelioma cell lines: MESO-7T, MESO-8T, MESO-12T, MESO-23T and MESO-29T [42] or MPM#2, MPM#26, MPM#26 and MPM#34 [43] were supplied by MesobanK [44] (MesobanK, Cambridge, UK). All cell lines were grown in RPMI-1640 Glutamax (Gibco, Waltham, MA, USA) with 10% foetal bovine serum (Gibco, Waltham, MA, USA) in a 5% CO_2_ humidified incubator at 37 °C. Growth media for cell lines designated MESO was supplemented with 20 ng/mL EGF (Peprotech, London, UK), 1 mg/mL hydrocortisone (Sigma, St. Louis, MO, USA) and 2 mg/mL heparin (Sigma, St. Louis, MO, USA). Cell lines were split every 3–4 days at approximately 80% confluency and grown for a maximum of 20 passages. All cell lines were authenticated by STR profiling and routinely confirmed as mycoplasma free.

### 2.2. Dual Labelling of Cell Lines

Mesothelioma cell lines were transduced with lentiviral particles carrying pHIV-Luc-ZsGreen (a gift from Bryan Welm; RRID: Addgene_39196). Lentiviral particle generation and cell line transduction were performed as previously described [45]. Transduction efficiency was assessed via fluorescence using a Nikon Eclipse Ti and CFI Plan-Fluor 10X (N.A.0.3) objective (Nikon, Tokyo, Japan).

### 2.3. In Vitro Bioluminescence Measurement

Dual labelled cells were seeded in 96 well plates (black walled, clear bottom; Corning, Glendale, AZ, USA; cat#3603) using a 1:2 serial dilution to give a range of 500 to 500,000 cells/well. The luciferase assay was performed after 4 h once cells had adhered to the plate. Media was replaced with 100 µL media containing 150 µg/mL luciferin (Promega, Madison, WI, USA; E1605). After 10 min incubation at room temperature, the bioluminescence signal was acquired using an IVIS Spectrum In Vivo Imaging System (Perkin Elmer, Waltham, MA, USA; Open filter, FOV = C, Imaging subject: in vivo). All bioluminescent signal was quantified using the Living Image Software (IVIS Imaging Systems, Perkin Elmer, Waltham, MA, USA).

### 2.4. Xenograft Generation

Fertilised Bovan Brown eggs (Henry Stewart Co., Ltd., Fakenham, UK) were incubated at 37 °C and 45% humidity (embryonic day 0; E0) in a specialised poultry incubator (Brinsea OvaEasy 380 Advance EX Series II Automatic Egg Incubator). Eggs were laid horizontally in incubation trays (Brinsea, Weston-super-Mare, UK), and the upward facing side marked to indicate the location for the window to be cut. For the duration of E0–E3, the incubator shelves were set to alternate tilting 45 degrees every 45 min. For *in ovo* experiments, E3 eggs were windowed by puncturing the wide base of the egg (air cell) with an egg piercer to remove about 5 mL of albumen with a 23 G needle, before sealing the hole with Nev’s Ink tape. Another hole was pierced on the labelled side of the egg, a 3 cm piece of 25 mm 3 M Scotch Magic invisible tape applied, and sharp scissor tips inserted into the pierced hole to carefully cut three sides (2 cm × 1 cm × 2 cm) of a rectangle to create a window in the eggshell. The fenestration area was sealed with approximately 4 cm of 25 mm 3 M Scotch Magic invisible tape, leaving a small tab to enable re-opening of the window, and eggs were placed back into the incubator until E7.

For *ex ovo* experiments, eggs were gently cracked at E3 and the contents transferred to UV-sterilised black weighing boats (Starlab, Hamburg, Germany). These were placed inside sterile 150 cm^2^ tissue-culture flasks with re-closable lids (Techno Plastic Products AG, Trasadingen, Switzerland) containing sterile water to maintain humidity and incubated until E7 in a Brinsea OvaEasy 360 incubator.

Prior to implantation on E7, cells were collected by trypsinisation, counted, washed in sterile phosphate-buffered saline (PBS), and pelleted via centrifugation. Experiments were optimised by implanting between 0.5 × 10^6^ and 2 × 10^6^ cells per egg in 10–15 µL; with 2 × 10^6^ cells implanted on a minimum of 12 eggs per cell line for the main experiments. Prior to adding the cells, the CAM was traumatised using a 1 cm wide strip of sterile gauze swab, causing a small bleed. The cells were directly pipetted onto this region of the CAM, without any support structure such as Matrigel or a silicon ring. Eggs were resealed and incubated until E14. Chick survival and tumour progression were monitored during experiments, with bioluminescence imaging (BLI) performed prior to fluorescence imaging and tumour dissection at E14.

### 2.5. In Vivo Bioluminescence Imaging (BLI)

Prior to dissection on E14, viability of tumours was routinely assessed by BLI with images acquired using an IVIS Spectrum In Vivo Imaging System (Open filter, FOV = C, Imaging subject: Other). For BLI, 250 µL of 15 mg/mL luciferin (Promega, Madison, WI, US; E1605) was injected into the yolk sac using a BD Micro-Fine 0.5 mL insulin syringe with 29 G needle (BD Biosciences, Franklin Lakes, NJ, USA), avoiding blood vessels. To determine the optimal post-injection timepoint for in vivo imaging, a chick with a visible tumour nodule at E14 was selected for each dual-labelled cell line and bioluminescence images acquired every 3 min for 2 h. A steady state maximal signal was reached by 45 min in each case, so this timepoint was used for all image acquisition. Where longitudinal BLI was carried out, the same procedure was followed on the indicated days. Bioluminescent signal was reported as total flux (radiance: p/sec/cm^2^/sr).

### 2.6. Fluorescence Imaging and Tumour Dissection

Following BLI at E14, tumours were imaged under a Leica M165FC fluorescence stereomicroscope with 16.5:1 zoom optics, fitted with a Leica DFC425 C camera (Leica Biosystems, Wetzlar, Germany). Tumours were imaged *in ovo* on the CAM and then dissected. Briefly, extra-fine straight-tip tweezers were utilised to manipulate the CAM to allow a sizeable circumference to be cut around the tumour nodules with spring bow micro scissors. Excised tumour nodules were placed in sterile PBS for *ex ovo* imaging. After removing any excess CAM, dissected tumours were weighed on a fine balance, and processed for immunohistochemical or transcriptional analyses. Following removal of the tumour, embryos were terminated in accordance with the UK Animals Scientific Procedures Act 1986 (amended 2012), under which the chick embryo is classified as non-protected until two thirds of gestation is reached at E14. No home office approval is required, and procedures were reviewed by the Liverpool Animal Welfare and Ethical Review Body.

### 2.7. Immunohistochemistry

Immediately after dissection tumour samples were placed in 1 mL 10% neutral buffered formalin (Sigma, St. Louis, MO, USA) for 16 h and then transferred to 70% ethanol. Following automated tissue processing, samples were embedded in paraffin blocks and sectioned at 4 µm onto SuperFrost Plus slides. Immunohistochemical staining was performed on the fully automated Leica BOND RX^M^. Primary antibodies: mouse anti-Pan cytokeratin (MNF116; Invitrogen, Walthan, MA, USA; MA1-26237 [human specific]; or AE1/AE3; Santa Cruz, Dallas, TX, USA; sc-81714 [human and chicken reactivity]) used at a dilution of 1:80 and pH9; rabbit anti-αSMA (Abcam ab5694 [human and chicken reactivity]) 1:200 and pH9; and mouse anti-Ki-67 (Novocastra, Newcastle-upon-Tyne, UK; NCL-L-Ki67-MM1) 1:200 and pH9. Antibody binding was visualised with diaminobenzidine (DAB), samples were counterstained with haematoxylin to assist in distinguishing between human tumour and chick cell nuclei, and sections were mounted with DPX mountant (Sigma, St. Louis, MO, USA). Images of the slides were acquired using digital slide scanners (Leica Aperio CS2 digital slide scanner or Zeiss Axioscan Z1).

### 2.8. Digital Image Analysis of Ki-67

Digitalised whole slide images of tumour nodules stained for Ki-67 were scored using open-source, digital pathology software QuPath version 0.2.0-m7 [46]. Briefly, 10 DAB-positive and 10 DAB-negative cells were manually selected as a training cohort to identify Ki-67 positive and negative cells, respectively. The tumour nodule was delineated manually using the wand tool prior to watershed nucleus detection (settings: requested pixel size 0.5 μm; background radius 8.0 μm; sigma 1.5 μm; min/max area 10/400 µm; threshold 0.1; maximum background intensity 2.0; and cell expansion 5 μm), which was optimised visually. The detection threshold value for Ki-67 positive cells (nucleus DAB OD mean) was manually set to 0.1 for all slides, following visual assessment. All measurements were exported into Excel to calculate the percentage of Ki-67 positive cells.

### 2.9. RNA Extraction and qRT-PCR

Tumours harvested from the CAM were rinsed in ice-cold PBS and transferred to RNAlater (Ambion, Life Technologies, Carlsbad, CA, USA). Total RNA was extracted as previously described [45] using a NucleoSpin RNA Tissue Kit (Macherey-Nagel, Dueren, Germany). For cell lines cultured *in vitro*, total RNA was extracted as previously described [47] using RNeasy columns (Qiagen, Hilden, Germany). Complementary DNA was reverse transcribed from 1μg RNA with RevertAid H-minus M-MuLV reverse transcriptase (Fermentas, Waltham, MA, USA) using an oligo-dT primer (Promega, Madison, WI, USA). qRT-PCR was performed using a CFX real-time PCR detection system (Bio-Rad, Hercules, CA, USA) and SYBR Green. Primer sequences were: *GAPDH* (for: 5′-CAATGACCCCTTCATTGACC-3′, rev: 5′-GACAAGCTTCCCGTTCTCAG-3′), *ACTB* (for: 5′-CACCTTCTACAATGAGCTGCGTGTG-3′, rev: 5′-ATAGCACAGCCTGGATAGCAACGTAC-3′), *MMP9* (for: 5′-TTCTGCCCGGACCAAGGATA-3′, rev: 5′-ATGCCATTCACGTCGTCCTT-3′) and *VEGF* (for: 5-CTCCACCATGCCAAGTGGTC-3′, rev: 5′-GCAGTAGCTGCGCTGATAGA-3′). Relative transcript expression is reported for genes of interest normalised to the mean values for *ACTB* and *GAPDH* (2^ΔCq^).

### 2.10. Analysis of CAM Vasculature

Microscopy RGB images of tumour nodules on top of the CAM were cropped to an area of approximately 1000 µm around the tumour nodule. A region of interest excluding the tumour was selected for analysis. The IKOSA CAM Assay (KML Vision, Graz, Austria) was used to determine the mean vessel thickness, as well as the total vessel area, total vessel length, and number of branching points normalised to the area analysed.

### 2.11. Magnetic Resonance Imaging (MRI)

MRI was performed using a horizontal bore 9.4 T Bruker Biospec 94/20 USR system (Bruker Scientific Instruments, Billerica, MA, USA). Following 10 min incubation at 4 °C, the egg was placed in a custom-built cradle, and an actively decoupled 3 cm diameter surface coil was affixed on the eggshell above the site of the tumour. An 86 mm volume coil was used for signal transmission, while the surface coil was used for signal detection. Initially scout images were acquired using a three-plane gradient echo sequence to localise the tumour. High resolution 3D images were subsequently acquired using a 3D TurboRARE (spin-echo, T2-weighted) pulse sequence with the following parameters: field of view 40 mm × 40 mm, matrix size 400 × 400 × 20, TR/TE = 1800/6.8 ms, effective TE: 80.74 ms, echo train length 8, slab thickness = 2 mm, averages 1, flip angle 90, scan time of approximately 8 min.

### 2.12. Statistical Analysis

Statistical analyses were performed in GraphPad Prism version 9 for Mac (GraphPad Software, San Diego, CA, USA). Distribution of data was assessed by Shapiro–Wilk test and data were analysed by parametric or non-parametric tests as appropriate. The number of independent samples, definition of error bars, and the statistical test employed are described in relevant figure legends. *p* values less than 0.05 were considered significant.

## 3. Results

### 3.1. Dual-Labelled MPM Cell Lines Efficiently Engraft on the CAM to Form Tumour Nodules

**Figure 1:**
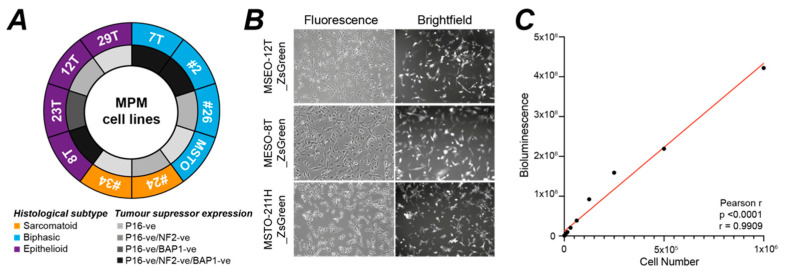
Characteristics of MPM cell lines used for engraftment. (**A**) Summary of the ten MPM cell lines used in this study. Histological sub-type was defined by the supplying cell bank, tumour suppressor status was inferred from immunoblotting [48]; (**B**) MPM cell lines were transduced with lentiviral particles carrying pHIV-Luc-ZsGreen. Example fluorescence images of MESO-12T, MESO-8T and MSTO-211H cells grown in vitro show high efficiency of transduction (*n* = 1), other cell lines in Figure S1A; (**C**) Example in vitro analysis of luminescence signal shows proportionality with cell number for the MESO-8T cell line (*n* = 1), supporting kinetics data in Figure S1B.

We selected ten MPM cell lines to test their establishment as CAM xenografts. The majority were quite newly established well-characterised early passage cell lines sourced through Mesobank [44], but we included the MSTO-211H cell line as a comparator that was established in long term culture several decades ago [41]. Together the ten cell lines represent the three major histological subtypes of MPM and common combinations of intersectional inactivation for the three key tumour suppressors (Figure 1A). All cell lines were dual-labelled with luciferase and ZsGreen, a human codon-optimised variant of *Zoanthus* sp. green fluorescent protein with bright fluorescence and high expression in mammalian cells. Labelling efficiency was around 90% in each cell line as assessed by fluorescence microscopy (Figure 1B and Figure S1A). Co-labelling with Firefly luciferase enabled BLI on addition of luciferin to measure cellular ATP as an estimate of viable MPM cell content within tumour nodules (Figure 1C and Figure S1B).

**Figure 2:**
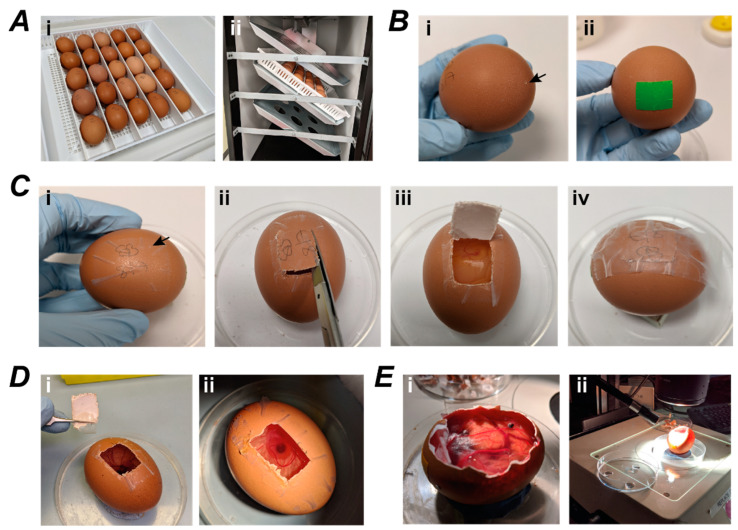
Workflow for MPM cell engraftment on the CAM. (**A**) Fertilised eggs are placed horizontally in white trays (i) and incubated at 37 °C to initiate embryonic development. Trays are rotated 45° every 45 min to prevent membranes sticking to the shell (ii); (**B**) Prior to windowing on embryonic day 3 (E3), a hole is made in the wide base of the egg (i; arrowhead) to allow 5 mL albumin to be removed and then sealed with Nev’s label tape (ii); (**C**) To create the window, a hole is pricked in the eggshell as a starting point (arrowhead) and a piece of Scotch tape placed over the area where the window is to be cut (i). Scissors are used to the cut the window (ii) leaving one side attached (iii). The window is then sealed shut with Scotch tape (iv); (**D**) Cells are implanted on E7 by removing the window (i) to expose CAM underneath (ii). Once cells have been added to the CAM, the window is sealed shut again (Civ); (**E**) At E14 the window is enlarged (i) to allow inspection of the embryo and imaging of tumour nodules (ii).

The methodology for establishment of MPM-xenografts is fully described in the methods section and illustrated in Figure 2. Briefly, to initiate development at E0 fertilised hen’s eggs were placed in an egg tray in a specialised poultry incubator (Figure 2A). On E3, a rectangular window was cut in the eggshell to make the CAM accessible and allow observation of tumour formation, progression, and embryo health (Figure 2B,C). This was reattached with tape (Figure 2Civ) and eggs immediately placed back into the incubator. The window was opened on E7 to check embryo survival and any dead or unfertilised eggs were discarded. Viable eggs with a properly developing embryo (Figure 2D) were used for implantation of MPM cells onto the CAM, then placed back into the incubator. On E14, eggs were examined under a fluorescence stereomicroscope to identify potential tumour formation. The use of ZsGreen-labelled cells allowed sensitive detection of even small tumour nodules, although in most instances MPM nodules were of substantial size and easily visible by eye (Figure 2E).

**Figure 3:**
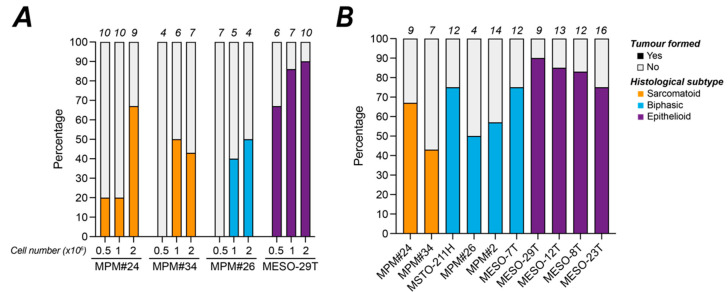
Efficiency of MPM cell line engraftment on the CAM. (**A**,**B**) Stacked histograms show the percentage of viable eggs at E14 where tumour nodules had engrafted. (**A**) Experimental determination of the optimal cell number for im plantation on the CAM for 4 cell lines; (**B**) Engraftment efficiency for the 10 MPM cell lines implanted with 2 million cells; supporting survival data in Table S1. The number of viable engrafted eggs at E14 for each cell line is shown above the bars on each graph. All experiments were *in ovo*.

**Figure 4:**
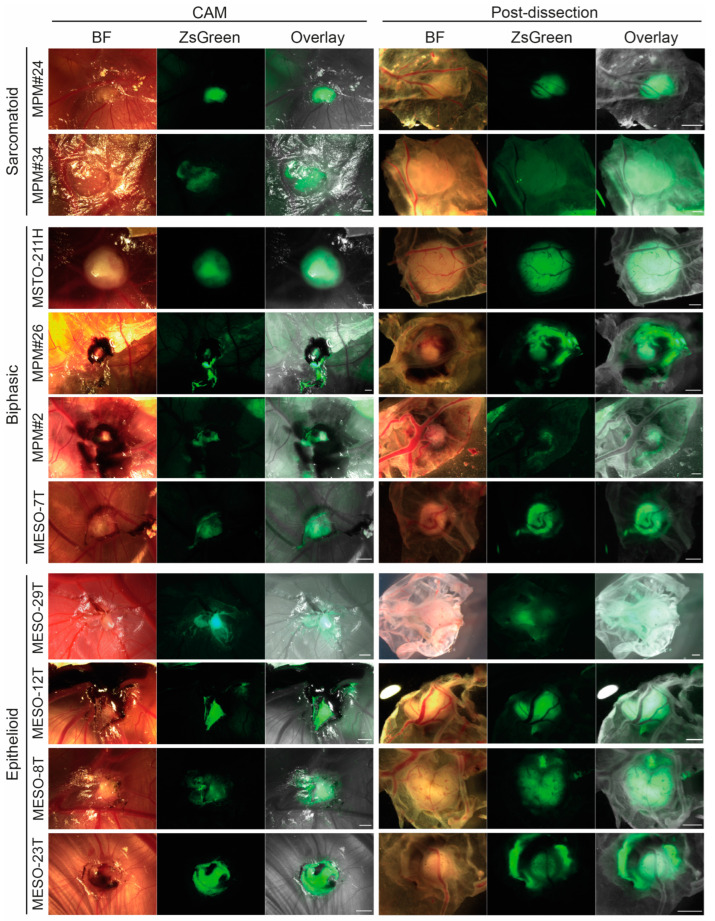
MPM cell lines all form vascularised tumour nodules on the CAM. Representative images of tumour nodules formed by each of the 10 MPM cell lines for the experiment shown in Figure 3. Nodules are shown in situ on the CAM *in ovo* (left) and post-dissection viewing the nodule from beneath (right). Scale bar 1 mm. BF, bright field.

We initially selected four cell lines to optimise the number of MPM cells for efficient *in ovo* implantation. In each case, 0.5, 1 or 2 million cells were implanted at E7, and the number of eggs with a visible tumour nodule and BLI signal was monitored at E14. Before taking experimental readings, the in vivo kinetics of xenograft bioluminescence was determined for each MPM cell line following injection of luciferin into the yolk sac (example in Figure S1); in every case steady state was reached by 45 min. At least 1 million cells were required for MPM#34 and MPM#26 to form nodules, whilst for most cell lines 2 million cells yielded the best engraftment on the CAM, producing more viable tumour nodules of a sufficient size for post-processing (Figure 3A). This protocol and cell number were therefore adopted for implantation of the other MPM cell lines, all of which we found could form vascularised tumours on the CAM, irrespective of histological subtype or genetic background (Figure 4). For embryos that survived until E14 (Table S1) engraftment rates, based on the formation of a viable 3D nodule, ranged from 43% to 85% and were highest for the epithelioid cell lines, which ranged from 75% to 85% with a mean nodule formation rate of 80% (Figure 3B).

### 3.2. Different MPM Cell Lines Establish Morphologically Distinct CAM Nodules That Exhibit Local Invasion and Recruit Chick Fibroblasts and Vasculature

The size and shape of tumour nodules varied between MPM cell lines (Figure 4). Nodules were examined in situ from above (overlay, third column), then dissected to view the underneath (overlay, sixth column). In general, the epithelioid cell lines formed larger nodules, whilst biphasic and sarcomatoid cells formed smaller nodules, with the notable exception of the very large nodules formed by the long established biphasic MSTO-211H cell line (Figure 4). Sarcomatoid nodules also appeared less well vascularised than those of other histopathological types.

**Figure 5:**
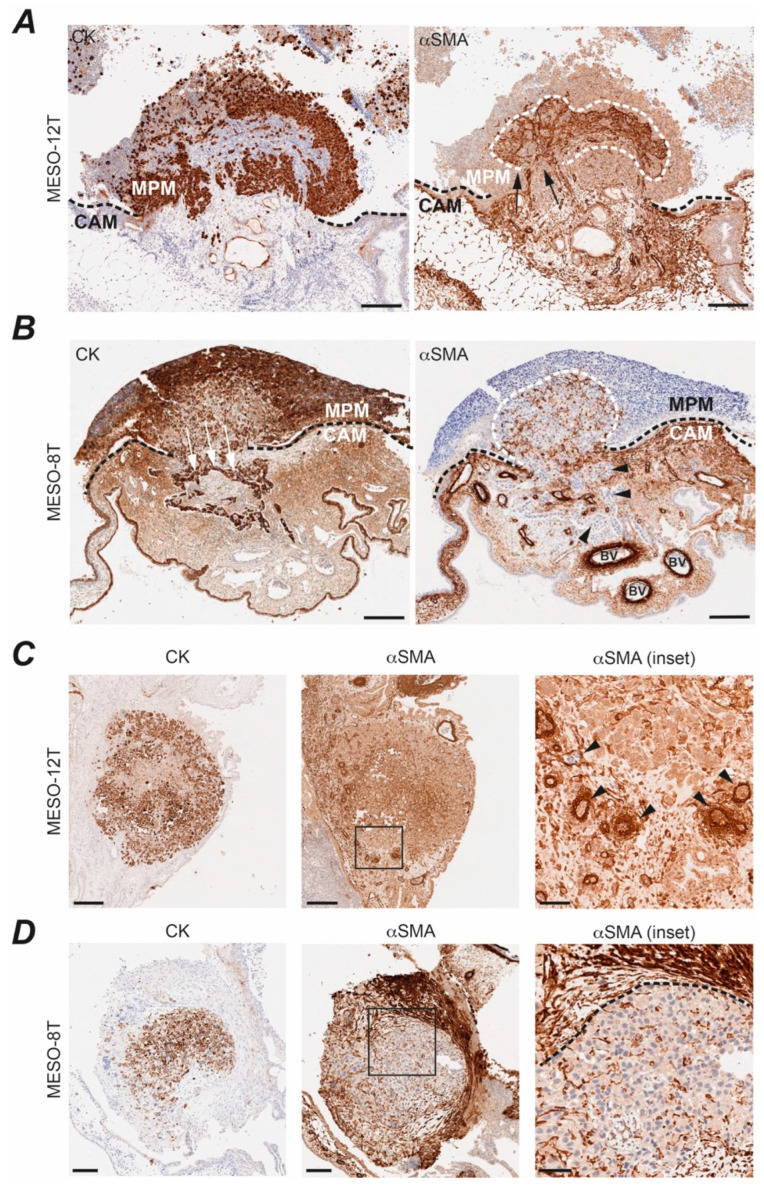
Histological characterisation of MPM CAM nodule morphology, local invasion, fibroblast recruitment and tumour vascularisation. (**A**) MESO-12T nodule sagittal section; CK (MNF116; left) and αSMA (right); scale bar 200 µm. Black dashed line, CAM and MPM tumour interface; white dashed line, region of infiltrating chick fibroblast-like cells within the tumour nodule. Black arrows, αSMA-positive cells moving into tumour nodule. Supporting IHC in Figure S2A. (**B**) MESO-8T nodule sagittal section; CK (sc-81714; left) and αSMA (right); scale bar 200 µm. Black dashed line, CAM and MPM tumour interface; white dashed line, region of infiltrating chick fibroblasts within the tumour. BV, αSMA-positive blood vessels; arrow heads, αSMA-negative MPM cells invading CAM. Supporting IHC in Figure S3A. (**C**) Tumour nodule vascularisation. MESO-12T nodule section; CK (MNF116; left) and αSMA (middle); scale bar 250 µm. αSMA inset (left); scale bar 50 µm. Black arrow heads, blood vessels. Supporting IHC for other markers in Figure S2B. (**D**) Fibroblast encapsulation. MESO-8T nodule transverse section; CK (MNF116; left) and αSMA (middle); scale bar 100 µm. αSMA inset (left); scale bar 50 µm. White line, fibroblast encapsulation; supporting IHC in Figure S3C. All experiments were *in ovo* and at least two nodules were examined per cell line.

Two epithelioid cell lines formed nodules with different gross morphology; MESO-12T (P16, NF2 altered) typically formed nodules above the CAM whilst MESO-8T (P16, NF2, BAP1 altered) nodules were often found beneath the CAM (Figure 4). We stained FFPE sections from the nodules to further explore their structure and composition (Figure 5). MPM cells are typically immunoreactive for pan-cytokeratin [49], staining for which clearly identified the MPM tumour cells and highlighted their morphological arrangement within the nodule. Sagittal sectioning through a MESO-12T nodule showed a raised spherical structure with densely packed tumour cells (Figure 5A and Figure S2A). In contrast, a MESO-8T sagittal section revealed a flat patch of cells having a large plane of contact with the underlying CAM, and local invasion of MESO-8T cells through the chorionic epithelium into the mesenchyme where a nodule of more loosely packed tumour cells formed (Figure 5B and Figure S3A). In both cases, cytokeratin positive tumours cells corresponded with regions that stained either positive or negative for nuclear BAP1 according to their BAP1 status (Figures S2A,B and S3C) and were intermingled with cells not stained for cytokeratin (Figure 5A,B).

Interaction of MPM cells with their microenvironment is crucial to progression of the human disease, where the cancer cells can instigate fibroblast infiltration [5]. We therefore wondered if the cytokeratin-negative cells within CAM nodules may be infiltrating chick fibroblasts. Indeed, profuse infiltration of fibroblast-like cells that stained strongly for αSMA was evident amongst the tumour cells above the CAM in sagittal sections of both MESO-8T and MESO-12T nodules (Figure 5A right, Figure 5B right). Similar intermingling of the two cell types were evident in transverse sections of additional nodules formed by these and other MPM cell lines (Figure 5C,D, Figures S3 and S8), with encapsulation of nodules by fibroblast-like cells sometimes observed (Figure 5D). In all cases, we saw mutually exclusive distribution of the strongly stained αSMA or pan-cytokeratin immunoreactive cells within the CAM nodules, with the strongly αSMA-positive cells displaying different morphology to the tumour cells. To rule out any possibility that these might be MPM cells that had undergone epithelial-mesenchymal transition (EMT), immunofluorescent staining was also performed on a frozen tissue section from MESO-8T nodule (Figure S4). The αSMA-positive cells exhibit the same morphology seen by IHC and are clearly distinct from the Zs-Green labelled MPM cells, confirming that they derive from the chick.

The chick vasculature is also immunoreactive for αSMA, revealing tumour-adjacent blood vessels within the CAM (Figure 5C, Figures S3A and S6A). Internal vascularisation was also seen within the MPM nodules evidencing tumour angiogenesis; the degree and phenotype of this intratumoural vasculature varied between cell lines and appeared particularly extensive within MESO-8T nodules (Figures S3A,B and S8).

### 3.3. MPM Tumour Nodules Express VEGF and Remodel the CAM Vasculature

**Figure 6:**
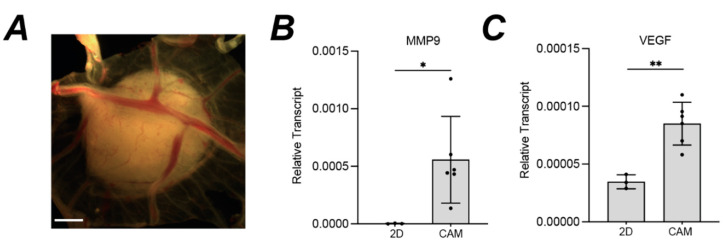
MPM tumour nodules increase transcription of invasive and angiogenic genes compared to 2D cultures. (**A**) Representative image of MSTO-211H tumour nodule used for RNA extraction, scale bar 1 mm; (**B**,**C**) qRT-PCR analysis comparing expression of *MMP9* (**B**) and *VEGF* (**C**) transcript levels in MSTO-211H grown as 2D in vitro cultures (*n* = 3) versus 3D CAM nodules (*n* = 6). Mean expression shown relative to the mean of *ACTB* and *GAPDH* (2^∆Cq^), error bars SD, unpaired *t*-test, * *p* < 0.05, ** *p* < 0.01. Supporting data for housekeeping genes in Figure S5. All experiments were *in ovo*.

Given the histological evidence for local invasion and tumour vascularisation, we wondered whether the microenvironment on the CAM stimulated an invasive and angiogenic transcriptional program in the MPM cells. RNA was extracted for qRT-PCR from MSTO-211H cells grown either in vitro in 2D, or *in ovo* as a 3D CAM xenograft. The C_q_ values for *ACTB* and *GAPDH* housekeeping genes were unaffected by the switch from 2D culture to 3D CAM (Figure S5). However, there was a significant increase in expression of both the metastasis-associated matrix metallopeptidase *MMP9*, and the growth factor *VEGF* that induces proliferation and migration of vascular endothelial cells (Figure 6).

**Figure 7:**
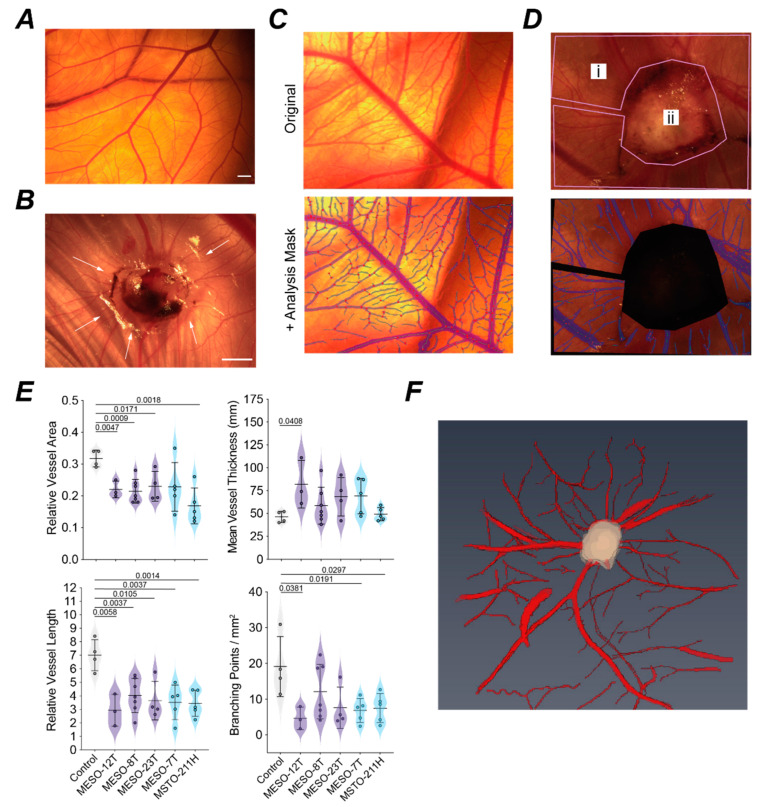
MPM tumour nodules remodel surrounding CAM vasculature. (**A**) Representative image of blood vessel branching on a normal non-tumour bearing CAM; scale bar 1 mm. (**B**) Radial remodelling of blood vessels (arrows) around an MPM nodule established by MESO-23T cells; scale bar 1 mm. (**C**,**D**) Example images of CAM vasculature for IKOSA analysis showing raw image (top) and analysis mask (below) for control non-tumour bearing CAM (**C**) and CAM vasculature around a MESO-7T nodule (**D**), (i) region used for vascular scoring, (ii) tumour nodule excluded. (**E**) Violin plots comparing characteristics of the CAM vasculature in non-tumour bearing CAM (control, *n* = 4) and in a 1 mm radius surrounding the tumour for MPM cell lines: MESO-12T (*n* = 3), MESO-8T (*n* = 7), MESO-23T (*n* = 4), MESO-7T (*n* = 5), MSTO-211H (*n* = 5). Histological sub-types: epithelioid (purple), biphasic (blue). Total vessel area and length are expressed relative to area of analysis (mm^2^). Normality was assessed by Shapiro–Wilk test and an unpaired *t*-test or Mann–Whitney test used as appropriate to compare tumour bearing CAMs to the control CAM group; *p* values indicate significant differences. (**F**) 3D rendered image derived from MRI analysis of the CAM vasculature around an MSTO-211H nodule. Supporting data in Figure S6B. All experiments were *in ovo*.

We also observed that MPM xenografts remodel the typical branching pattern of the CAM vasculature into a radial pattern of vessels recruited into the nodule (Figure 7A,B). IKOSA CAM assay software was used to create an analysis mask to quantify parameters for vessels on the control CAM or immediately adjacent to the xenografts (Figure 7C,D). On normalisation to the analysis area, the total vessel lengths measured on tumour bearing CAM were significantly lower than for the control CAM (Figure 7E). This was generally associated with decreased total vessel area and the density of branching points around MPM CAM xenografts and a trend towards increased vessel thickness (Figure 7E). Thus, MPM xenografts typically remodel the surrounding CAM vasculature to recruit large, less branched feeder vessels. This 3D vascular network around the xenograft can be visualised by MRI (Figure 7F and Figure S6B). Intratumoural vascularisation shows branching of these feeder vessels occurs within MPM nodules to sustain the xenografts (Figure 5C, Figure S3A and S8).

### 3.4. Bioluminescence Imaging Estimates MPM Tumour Burden and Viability

**Figure 8:**
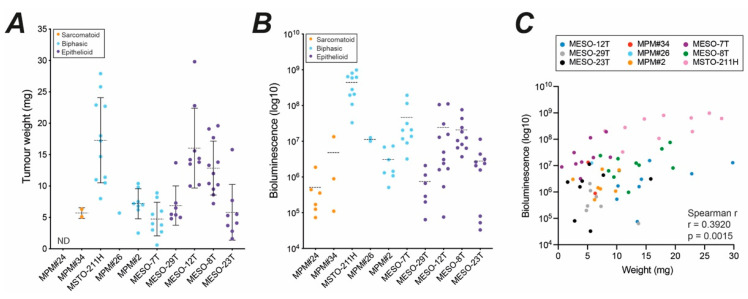
Bioluminescence imaging reliably estimates tumour burden. (**A**) Plots summarising measurement of individual tumours at E14 that were established from 2 million cells for each MPM cell line: tumour weight. MPM#34 *n* = 2, MSTO-211H *n* = 11, MPM#26 *n* = 1, MPM#2 *n* = 8, MESO-7T *n* = 9, MESO-29T *n* = 7, MESO-12T *n* = 8, MESO-8T *n* = 11, and MESO-23T *n* = 8. Dotted lines indicate the mean value for each cell type and error bars indicate standard deviation. ND, not done. (**B**) Plot summarising bioluminescence signal of individual tumours at E14. MPM#24 *n* = 6, MPM#34 *n* = 3, MSTO-211H *n* = 10, MPM#26 *n* = 2, MPM#2 *n* = 7, MESO-7T *n* = 9, MESO-29T *n* = 6, MESO-12T *n* = 12, MESO-8T *n* = 10, and MESO-23T *n* = 10. Dotted lines indicate the mean value for each cell type. (**C**) Positive correlation between bioluminescence signal (panel B) and dissected tumour weight (panel A); Spearman *r* = 0.39, *p* = 0.0015, *n* = 63. Correlation for individual cell lines in Figure S7. All experiments were *in ovo*.

The CAM xenografts exhibit size, architecture and microenvironment phenotypes that are in part defined by the MPM cell line. However, all cell lines can form vascularised nodules within 7 days that exhibit some fibroblast-like infiltrate, reflecting key aspects of the human disease and supporting utility of the model for testing therapeutic interventions. We therefore evaluated protocols to facilitate quantitative assessment of tumour burden and viability. For each MPM cell line, engrafted nodules established from 2 million cells were subject to BLI at E14 to assess tumour viability before dissecting the nodules away from the CAM and weighing (Figure 8). The mean tumour weight for many MPM cell lines was between 5 mg and 7 mg, whilst nodules formed by MESO-8T, MESO-12T and MSTO-211H were substantially larger with mean weights between 15 mg and 17 mg (Figure 8A) reflecting visual assessment (Figure 4).

**Figure 9:**
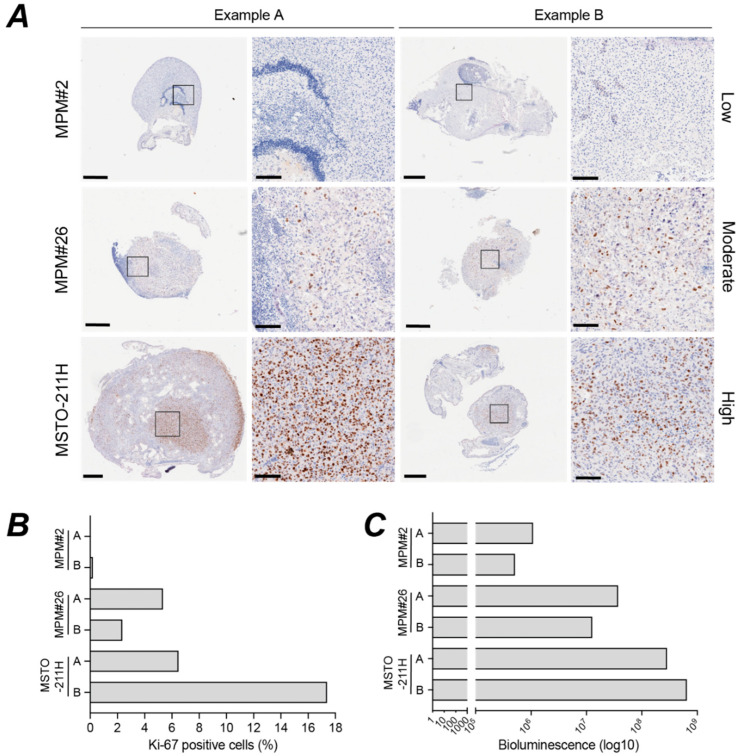
Bioluminescence signal corresponds closely with Ki-67 staining for proliferating cells. (**A**) Ki-67 staining of two independent MPM nodules for each of three MPM cell lines chosen as examples of low, moderate, and high staining. Whole tumour nodules (left, scale bar 500 µm) and higher magnification of regions (right, scale bar 100 µm). Supporting IHC for other markers in Figure S8. (**B**) Ki-67 score determined by Qupath analysis of images show in A (*n* = 2). Supporting data in Figure S9A. (**C**) Corresponding bioluminescence signal for the tumours shown in A (*n* = 2). Pearson correlation between Ki-67 score and bioluminescence, *r* = 0.96, *p* = 0.0023. Supporting data in Figure S9B. All experiments were *in ovo*.

BLI measurements exhibit a much larger dynamic range (Figure 8B) but reflect the trends for tumour weight. Interestingly, nodules established by biphasic MPM had proportionally higher mean BLI readings relative to tumour weight than epithelioid nodules. As BLI signal is proportional to cellular ATP, this may suggest a generally higher metabolic rate in biphasic compared to epithelioid CAM xenografts. Alternatively, epithelioid nodules may have greater infiltration of chick stromal cells that contribute to tumour weight but not BLI signal. Despite this, comparing individual xenografts across all the cell lines, BLI provided a reasonable estimate of MPM tumour burden compared to tumour weight (Figure 8C, Spearman *r* = 0.39, *p* = 0.0015). This relationship appeared more robust in some cell lines than others (Figure S7), again likely reflecting the degree to which individual MPM cell lines instigate chick cell infiltration (Figure 5) and their proliferative capacity on the CAM (Figure 9). Therefore, in addition to being very sensitive and relatively high throughput, BLI can provide additional information to interpret MPM xenograft growth or regression, especially if used longitudinally and in combination with end-point histological markers to assess nodule composition and proliferation.

Immunohistological staining for Ki-67 is widely used to estimate the proliferative index for FFPE tissue [50]. CAM nodules established from different MPM cell lines exhibit large differences in the percentage of tumour cells staining positive for Ki-67 at E14 (Figure 9A, Figures S2, S3 and S8). Intriguingly, despite forming viable nodules two BAP1 negative cell lines, MESO-8T and MPM#2, had low Ki-67 staining, potentially indicating they spend longer in G0/G1 [51]. As the Ki-67 antibody did not stain chick cells, and we wanted to compare whole nodule measurements of weight, BLI and Ki-67 staining, QuPath was trained to derive the percentage of Ki-67-positive cells within representative nodules derived from three biphasic MPM cell lines (Figure 9B and Figure S9). We compared the MPM#2 xenografts (P16, NF2 and BAP1 altered) that had a very low percentage of tumour cells staining positive for Ki-67, to MPM#26 xenografts (P16 and NF2 altered) which stained moderately for Ki-67, and MSTO-211H xenografts (P16 altered) that had an extremely high frequency of Ki-67 staining. Importantly, the Ki-67 scores were highly correlated with BLI measurements for nodules from these cell lines (Figure 9C, Pearson *r* = 0.96, *p* = 0.0023), reinforcing the utility of BLI as a live measurement for CAM xenografts that reflects not only tumour size but also tumour cell content and proliferative capacity. Considering the higher sensitivity of the BLI assay it may have utility even in less proliferative nodules where Ki-67 is hard to score.

**Figure 10:**
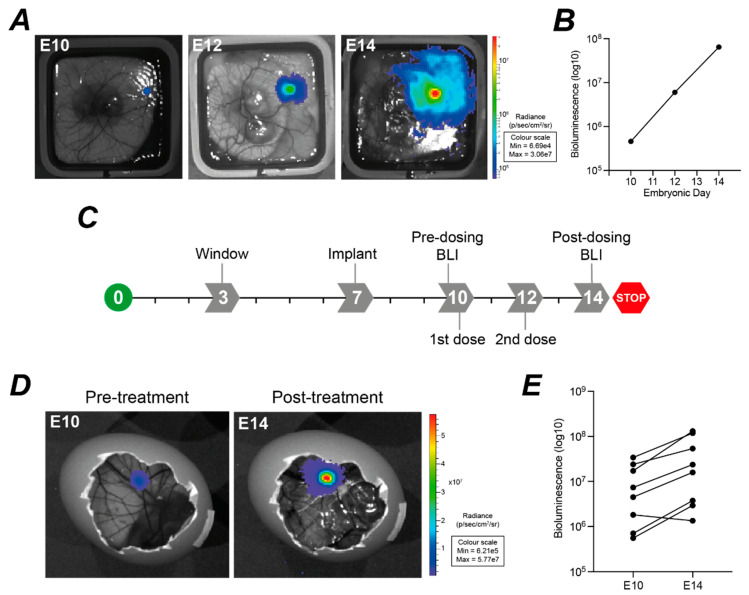
Bioluminescence imaging can be used to measure longitudinal responses. (**A,B**), Example of bioluminescence signal at E10, E12 and E14 monitoring *ex ovo* growth of an untreated MESO-8T tumour nodule. (**C**) Proposed experimental timeline for BLI imaging to evaluate xenografts pre- and post-dosing. (**D,E**) Comparison of bioluminescence signal for *in ovo* MSTO-211H tumour nodules at E10 and E14, following two yolk sac injections of PBS at E10 and E12 (*n* = 8).

Given the variability in both tumour weight and BLI signal for xenografts established from 2 million cells of any given cell line (Figure 8A,B), we investigated the possibility of using longitudinal BLI to monitor nodules over time. Measurements can be taken at E10 and subsequently at two-day intervals to generate growth curves (Figure 10A,B) although regular handling of the eggs with repeated luciferin injection into the yolk sack may reduce embryo survival. Mesothelioma is relatively resistant to standard chemotherapy, with cisplatin/pemetrexed showing only very modest clinical benefits and limited activity in vivo even with extended dosing [52,53]. While we have previously shown activity for cisplatin in CAM xenografts for a breast cancer cell line, our preliminary experiments showed no demonstrable activity in mesothelioma CAM xenografts. To develop a protocol for use in therapeutic testing, where eggs also need to be handled to dose with drugs, we therefore trialled an experimental timeline with vehicle control only (Figure 10C). PBS was injected into the yolk sac of established MSTO-211H xenografts at E10 and E12. Two BLI measurements were taken, at E10 pre-treatment, and at E14 post-treatment (Figure 10D). In this experiment, there was 80% survival between E10 to E14, and in 7 tumours the BLI signal increased by a mean of 4.40-fold (SD 1.82). Designing strategies using pre- and post-dosing BLI could reduce the effect of inter-egg variability on estimation of tumour burden, and so reduce the number of chick embryos required to fully power studies in line with 3Rs principles.

## 4. Discussion

MPM remains an area of critical unmet clinical need, despite a growing understanding of the processes driving its development and spread. Capitalising on this is unfortunately slow and costly, with drug development often taking 10 years or more from concept to clinical application, and with an estimated cost of around a billion dollars in research costs for each drug entering practice. Research performed using cultured cell lines, or rodent models, has historically corresponded poorly to outcome in eventual clinical trials. Thus, a continuum of preclinical models is required to embody different aspects of a human disease to screen and validate drug responses. Here, we describe protocols and analysis for a novel 3Rs-compliant CAM model for MPM that is rapid, economical, scalable, and adaptable, and which covers the spectrum of MPM histopathological types and common genetic alterations. It provides a wide range of in vivo readouts of tumour biology and viability within days rather than months lending itself to development as a useful preclinical model.

The CAM proved to be a conducive environment for MPM, as all 10 cell lines that we tested engrafted very effectively, particularly the epithelioid MPM where, on average, viable nodules formed in 80% of cases. As the CAM model is very economical compared to in vivo approaches in rodents, this facilitates use of a sufficient number and diversity of low passage cell lines to capture some of the heterogeneity of MPM and encompass the common genetic changes. For future evaluation of therapeutics, this could facilitate identification of relatively small subgroups that are likely to respond and may prevent discard of viable compounds. The diverse readouts available for CAM xenografts can be tuned depending on the experimental question, enabling specific hallmarks of cancer to be monitored, for example when assessing anti-proliferative, anti-invasive or anti-angiogenic therapeutic compounds.

Xenografts are less able to recapitulate the histology of the original tumour when derived from cell lines established in 2D culture, compared to fresh patient derived samples, whatever the host organism. However, the CAM model enables MPM cell lines to adopt a 3D architecture, which is characteristic for each cell line and partly addresses the importance of the tumour microenvironment. We observed pronounced interaction of MPM tumour nodules with chick fibroblasts as well as the chick vasculature. Comparing MSTO-211H xenografts in SCID mice [5] with MSTO-211H CAM xenografts shows the same diffuse infiltration of morphologically similar αSMA-stained fibroblast-like cells (Figure S8). This could therefore provide a more holistic model to assess drug responses and potentially facilitate testing of therapeutics targeting fibroblast and MPM interactions, or tumour angiogenesis, which are less easily modelled *in vitro*. For example, CAFs in MPM produce CTGF, which promotes MPM growth and correlates with survival outcomes, providing a potential therapeutic target [4,54]. The VEGF inhibitor bevacizumab improves chemotherapy outcomes for some patients [8] and, although other anti-angiogenic therapies have not been successful in trials, there remains an interest in targeting abnormal tumour vasculature in MPM [55].

Like immunodeficient mouse models, at early stages of embryonic development the chick lacks a fully functioning immune system which facilitates engraftment of human tumour cells on the CAM. The chick immune system develops during the period when tumour nodules grow, and by E18 fully immunocompetent chick lymphocytes can be detected [56]. However, in the UK non-protected models are terminated at E14 and, although immune cells including macrophages and lymphocytes are observed earlier in development [56], their presence in CAM xenografts at E14 has not been reported. Thus, in common with rodent flank xenografts in non-humanised models, CAM xenografts of cancer cell lines cannot be used to test immune modulators or inhibitors at E14. There is however potential to further increase the complexity of the CAM tumour microenvironment, by co-culture of MPM cells with autologous or heterologous human immune cells to investigate cellular interactions and potentially allow limited assessment of the effects of drugs on this interaction.

One challenge in fully realising the potential of CAM models in assessing drug responses is selecting the best methodology to evaluate tumour growth or regression. Tumour dimensions may be estimated, or excised nodules weighed, however these methods have limitations. Using dual-labelled MPM cell lines allowed tumours to be monitored by both fluorescence and bioluminescence, and we found the latter invaluable in distinguishing viable tumour nodules that were fully engrafted and vascularised during the experimental timeline. Although the utility of BLI could potentially be limited by the transduction efficiency, our experience is that many cell types transduce with high efficiency, as was the case with all the MPM cell lines tested. The reliance of BLI on ATP makes it superior to fluorescence imaging as BLI only detects metabolically active tumour cells, whilst the reliance on transduced luciferase ensures that only tumour cells and not chick cells are quantified. The latter is particularly important for MPM CAM xenografts, where we saw substantial infiltration of fibroblast-like chick cells. Importantly, whilst the BLI signal showed moderate correlation with tumour weight, we found a high correlation of BLI with Ki-67 staining as an endpoint measure of viable tumour cells. BLI methods have been published for other CAM xenografted cancer cell types, for example urothelial carcinoma [39] and pancreatic ductal adenocarcinoma [37]. However, our methodology differs in using yolk sac injection of luciferin, rather than topical application, to provide reproducible delivery into viable vascularised tumours and facilitate longitudinal measurement of tumour viability.

In other studies, CAM xenografts have been successfully treated with drugs administered by topical application [37], intravenous injection [57], or allantoic/yolk sac injection [40]. The choice of administration route is in part influenced by the nature of the drug and any delivery vehicle. Whilst topical application is simple, it is less amenable to accurate dosing. Intravenous injection provides direct administration of drug via the tumour vasculature, but is technically challenging and may preclude multiple dosing, whilst allantoic/yolk sac injection is an easier route to enable drug delivery via the tumour vasculature that is more amenable to repeat dosing. Here, for the purposes of establishing an experimental timeline, we demonstrate yolk sac administration of both luciferin for BLI monitoring and double injection of a vehicle treatment. Using pre- and post-dosing BLI can substantially reduce the effect of inter-egg variability on estimation of tumour response to drugs, and so reduce the number of chick embryos required to fully power studies in line with 3Rs principles. Further studies will be required to refine drug delivery approaches using agents with greater activity in MPM.

While the MPM-CAM xenograft model cannot fully replace rodent or other higher organismal model systems, it provides a complementary 3Rs compliant model to study tumour biology that we believe will in future allow more efficient screening of targets and help identification of subgroups more likely to benefit from therapy. Our studies suggest that certain MPM cell lines with highly responsive BLI signals may be most amenable for testing anti-proliferative therapies, including MESO-7T, MESO12-T and MSTO-211H. In contrast, for anti-invasive therapies histological analysis of MESO-8T nodules would be preferable, whilst many cell lines appear appropriate for evaluating anti-angiogenic therapies using IKOSA CAM analysis combined with immunohistochemistry. Furthermore, these initial MPM-CAM xenograft models for cultured MPM cell lines open the door to ongoing development that can incorporate further aspects of the MPM tumour microenvironment and architecture, for example by co-engrafting MPM cell lines together with human fibroblasts and/or immune cells, and engrafting patient-derived cells or tissue explants coupled with alternative preclinical imaging methods.

## 5. Conclusions

MPM-CAM xenografts can be efficiently established using MPM cell lines derived from tumours with a range of histopathological sub-types and tumour suppressor inactivation. These xenografts can mimic stromal and vascular interactions of clinical MPM lacking in 2D and most 3D cell cultures. Bioluminescence imaging can be readily used to evaluate MPM tumour burden over time, and is readily combined with multimodal imaging, transcriptional and histopathological analysis to determine tumour size, vascularisation, invasion, stromal composition, and proliferative capacity. The MPM-CAM model could therefore provide an invaluable component of the drug development pathway for MPM, and the methods for combined readouts could be extrapolated for use in multiple cancer types.

## Data Availability

All data associated with the study are included in this manuscript or its Supplementary Files.

## Supplementary Materials

The following supporting information can be downloaded at: https://www.mdpi.com/article/10.3390/cancers14235836/s1, Supplementary methods; Figure S1: Dual labelling of MPM cell lines and *in ovo* kinetics for bioluminescence imaging (BLI); Figure S2: Additional MESO-12T CAM nodule immunohistochemistry and histology; Figure S3: Additional MESO-8T CAM nodule immunohistochemistry and histology; Figure S4: Anti-alpha-SMA immunofluorescence on MESO-8T CAM nodule. Figure S5: Cq values for the housekeeping genes actin and GAPDH are not affected by 3D growth on the CAM; Figure S6: Additional modalities for MPM-CAM xenograft and vasculature analysis; Figure S7: Tumour weight versus bioluminescence signal for individual MPM cell lines; Figure S8: Additional MPM#2, MPM#26 and MSTO-211H CAM nodule immunohistochemistry; Figure S9: QuPath analysis of Ki-67 staining; Table S1: Viability at E14 for eggs engrafted with MPM cells at E7.
