# Supplementary material for: The Chick Embryo Xenograft Model for Malignant Pleural Mesothelioma: A Cost and Time Efficient 3Rs Model for Drug Target Evaluation
Source: Cancers (Basel). 2022 Nov 26;14(23):5836. doi: 10.3390/cancers14235836 (PMC9740959; doi:10.3390/cancers14235836)
Supplement: Supplementary file 1 [file cancers-14-05836-s001.zip › cancers-2056424-supplementary.pdf]

Supplementary Methods

Immunofluorescence

Immediately after dissection tumour samples were washed in sterile PBS and placed in 1 ml 4% paraformaldehyde (Sigma) for a maximum of 12 h. Following fixation, the samples were transferred to 6% sucrose for 16 h, then 12% sucrose for 24 h and finally into 20% sucrose for 24 h. Following sucrose infiltration, samples were placed in T-12 Peel-A-Way embedding moulds (Polysciences), covered with Shandon Cryomatrix (Thermo Scientific) and set by placing the mould on dry-ice for 10 min. 10μm sections were cut using a cryostat (Leica) and collected onto SuperFrost Plus™ slides (Thermo Scientific). Prior to immunofluorescent staining, slides were thawed at room temperature and a hydrophobic Liquid Blocker Super PAP pen used to create a boundary. Firstly, samples were rehydrated with PBS for 10 min, incubated with 50 mM NH_4CL for 20 min and then blocked for 30 min (blocking buffer: 1% bovine serum albumin, 0.1% Triton X-100, 0.4% Tween20 in PBS). Slides were incubated overnight at 4°C with 100µl primary antibody (rabbit anti-αSMA; Abcam #ab5694; 1:200 diluted in blocking buffer) in a liquid chamber. Samples were washed 3 times with blocking buffer and incubated for 1 h at room temperature in the dark with secondary antibodies (Goat Anti-Rabbit Alexa Flour 594; Invitrogen #A-11012; 1:500 in blocking buffer). Following two washes with PBS and one wash with dH_2O, coverslips were mounted using Mowiol (Sigma) supplemented with DAPI (1:10,000; Invitrogen). Images were acquired using a Nikon Eclipse Ti and CFI Plan-Fluor 10X (N.A.0.3) or CFI Super Plan Fluor 40XC (N.A. 0.60) objective.

Immunohistochemistry

Additional IHC antibody conditions: mouse anti-BAP1(C-4; Santa Cruz sc-28383) 1:250 and pH9. Control staining was performed alongside: no antibody (antibody diluent only) and mouse IgG1 negative control (DAKO #X0931).

Supplementary Figures

Figure S1. Dual labelling of MPM cell lines and *in ovo* kinetics for bioluminescence imaging (BLI).
Figure S2. Additional MESO-12T CAM nodule immunohistochemistry.
Figure S3. Additional MESO-8T CAM nodule immunohistochemistry.
Figure S4. Anti-αSMA immunofluorescence on MESO-8T CAM nodule.
Figure S5. Cq values for housekeeping genes *A CTB* and *GAPDH* are not affected by 3D growth on CAM.
Figure S6. Additional modalities for MPM-CAM xenograft and vasculature analysis.
Figure S7. Tumour weight versus bioluminescence signal for individual MPM cell lines.
Figure S8. Additional MPM#2, MPM#26 and MSTO-211H CAM nodule immunohistochemistry.
Figure S9. QuPath analysis of Ki-67 staining.

Supplementary Tables

Table S1. Viability at E14 for eggs engrafted with MPM cells at E7**.**

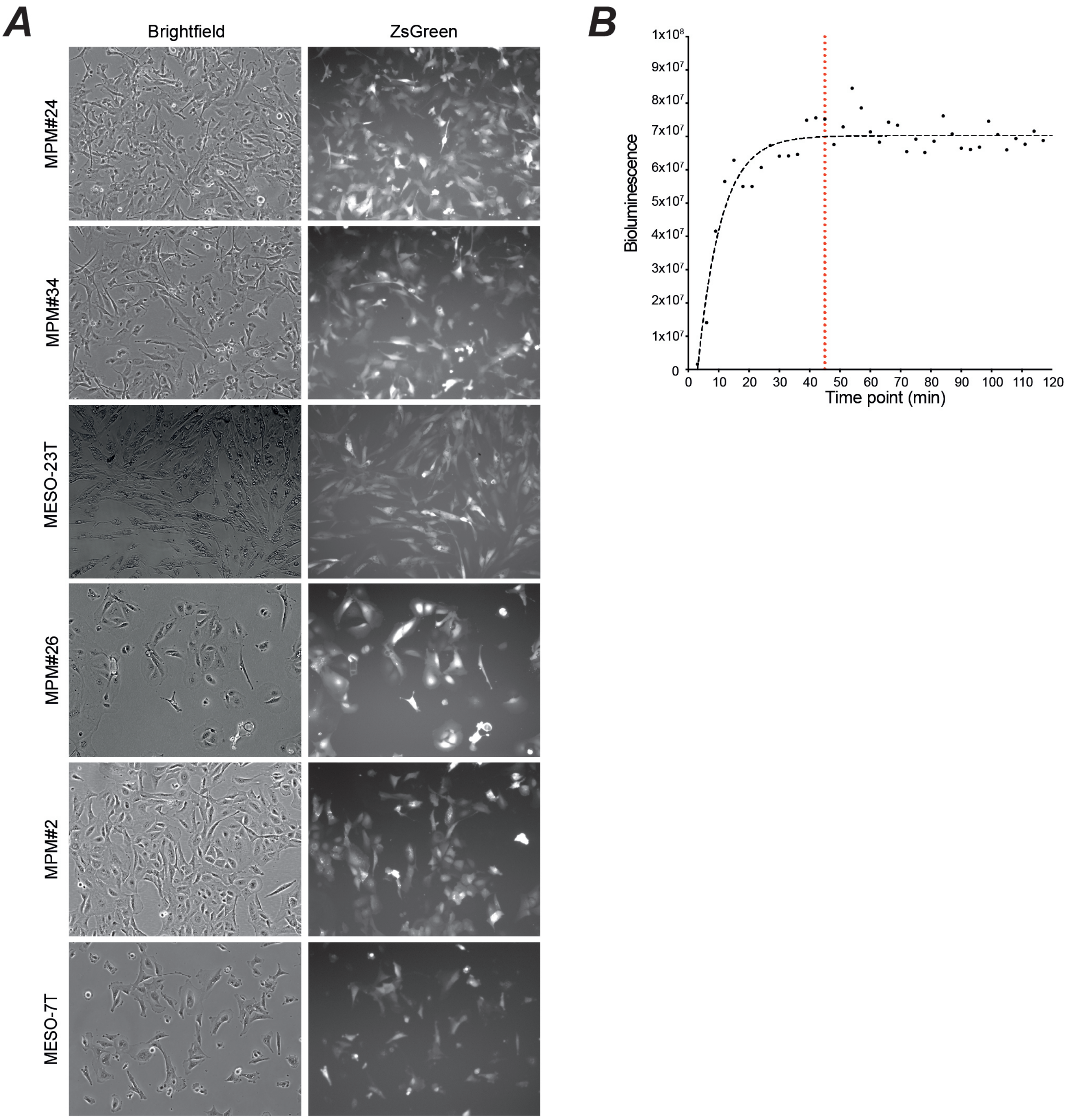

Figure S1. Dual labelling of MPM cell lines and *in ovo* kinetics for bioluminescence imaging (BLI). ***A,*** *In vitro* fluorescent microscope images of dual-labelled MPM cell lines. ***B,*** Example of *in ovo* kinetics for BLI signals recorded from an engrafted MESO-8T nodule over 2 hours following yolk sac injection of luciferin. A steady state plateau in BLI signal was reached by 45 min for all MPM cell line nodules.

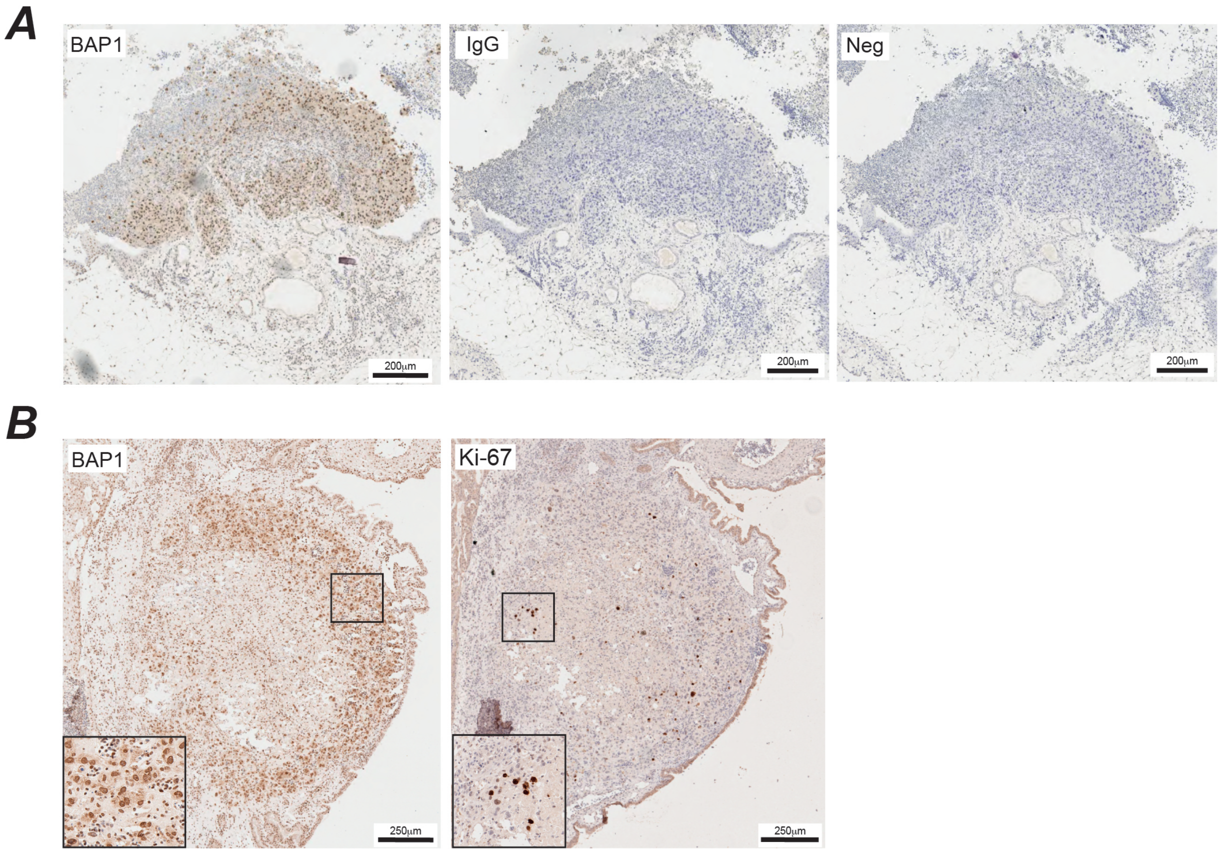

Figure S2. Additional MESO-12T CAM nodule immunohistochemistry.
Additional staining on adjacent sections for the examples of MESO-12T nodules shown in Figure 5A (***A***) and Figure 5C (***B***). Stain and scale are indicated on each image. MESO-12T are positive for nuclear BAP1 and proliferating cells are stained with Ki-67. Control staining was performed in parallel with an IgG negative control (IgG) or antibody diluent only (Neg). Inset images highlight BAP1 nuclear positive and Ki-67 positive tumour cells.

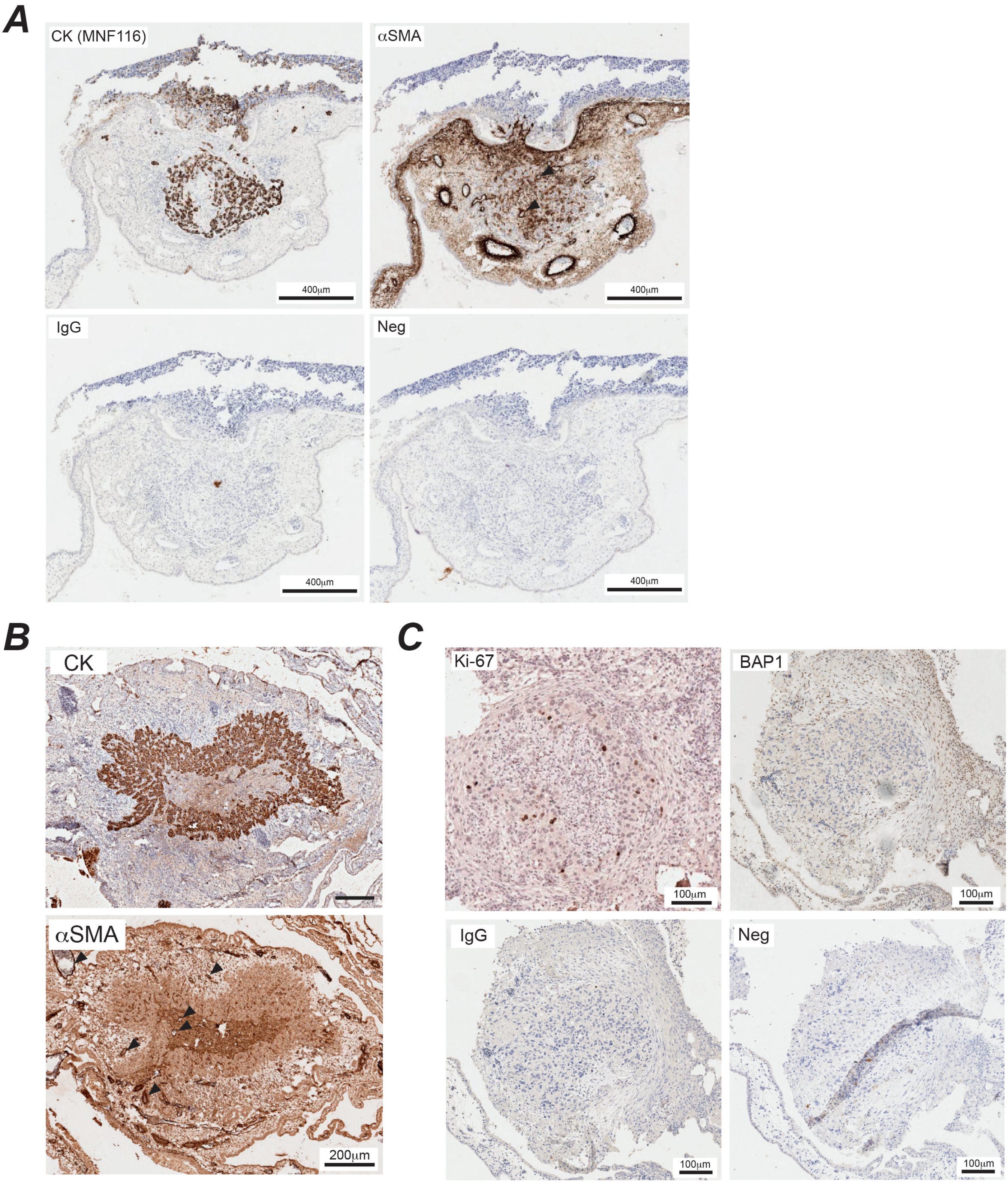

Figure S3. Additional MESO-8T CAM nodule immunohistochemistry

Additional staining on adjacent sections for the examples of MESO-8T nodules shown in Figure 5B (***A***) and Figure 5D (***C***). ***B,*** Example of cytokeratin and αSMA from a third MESO-8T nodule. Stain and scale are indicated on each image. MPM cells are stained with pan-cytokeratin (CK), chick fibroblasts and blood vessels are stained with αSMA, proliferating cells are stained with Ki-67, and MESO-8T are negative for nuclear BAP1. Control staining was performed in parallel with an IgG negative control (IgG) or antibody diluent only (Neg). Arrowheads indicate small blood vessels.

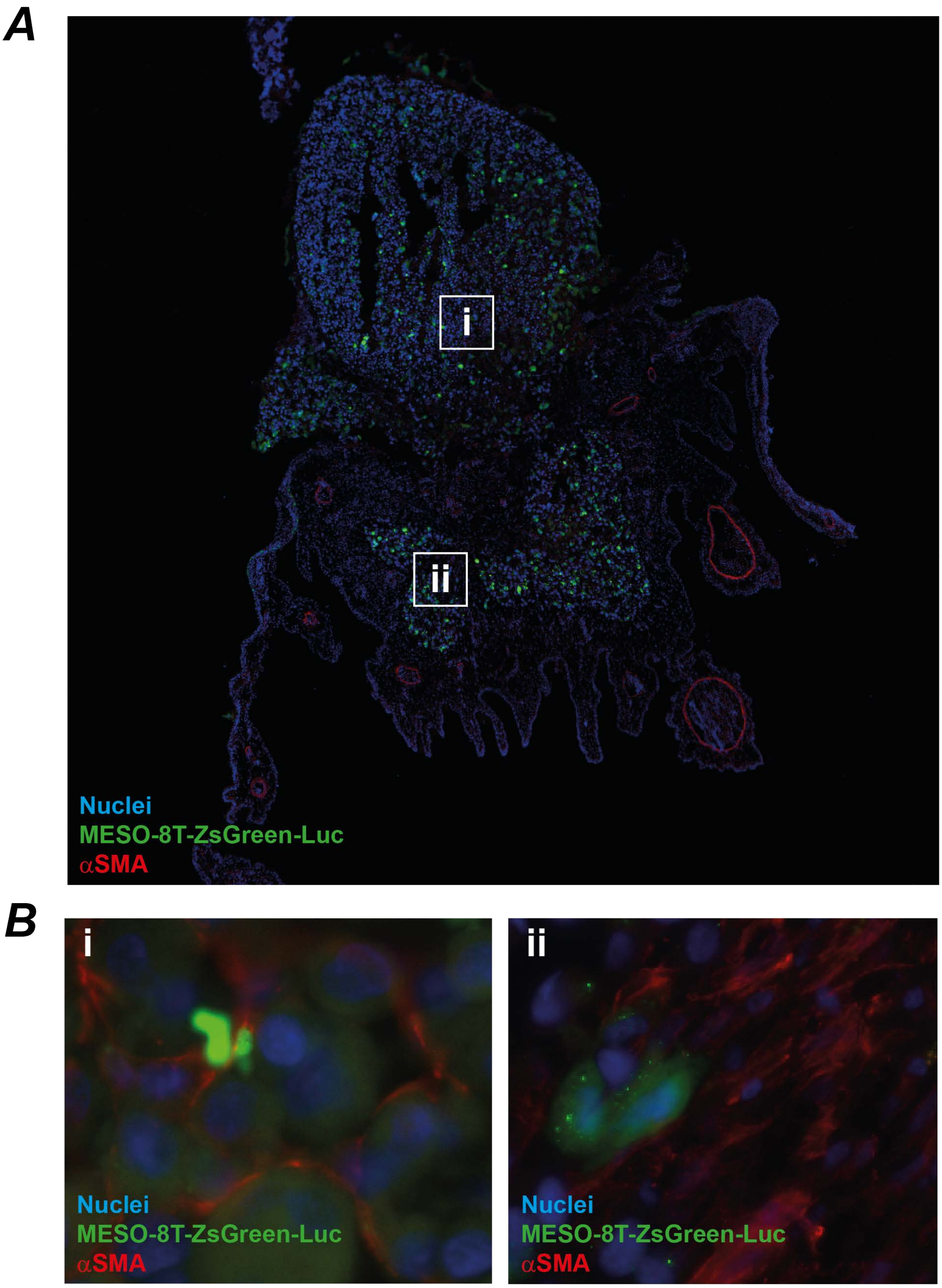

Figure S4. Anti-αSMA immunofluorescence on MESO-8T CAM nodule. Immunofluorescent staining was performed on frozen sections from a MESO-8T CAM nodule. Whole sample (***A***) and inset regions (***Bi,*** within tumour nodule; ***Bii***, within CAM). Staining of αSMA positive cells (red) is mutually exclusive from ZsGreen expressing MESO-8T cells (green). Nuclei (blue) of human and chick cells were stained using DAPI.

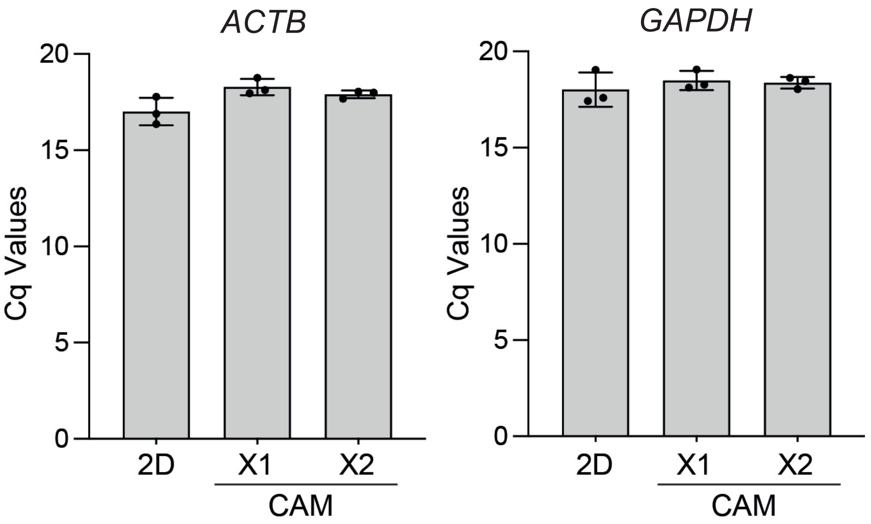

Figure S5. Cq values for the housekeeping genes *ACTB* and *GAPDH* are not affected by 3D growth on the CAM.

qRT-PCR was used to compare Cq values for *ACTB* or *GAPDH* expression in MSTO-211H cells grown in 2D culture *in vitro* (n=3) with those for cells grown in 3D culture in vivo on the CAM (2 independent experiments X1 and X2, each with 3 nodules). These data were used in calculation of the relative expression for other transcripts shown in Figure 6.

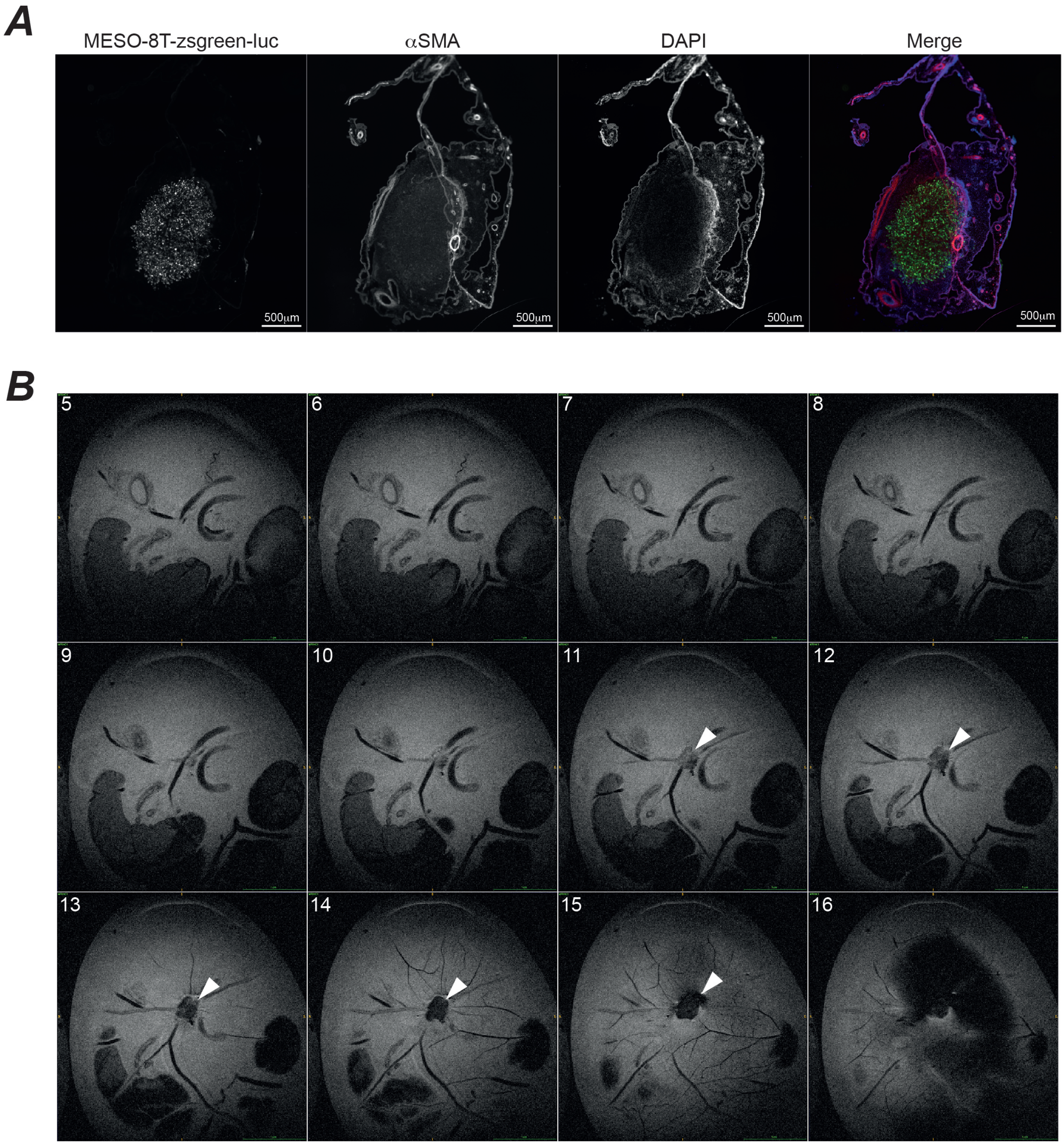

Figure S6. Additional modalities for MPM-CAM xenograft and vasculature analysis.

A, Immunofluorescent staining on a frozen section of MESO-8T tumour nodule, showing tumour adjacent blood vessels stained by αSMA. ***B,*** Representative seqential MR images in the coronal plane from the 3D dataset. The 3D data set covered a volume of interest of 40mm X 40mm X 2mm and was focused on the CAM membrane where the tumour was located. The number corresponding to each slice is given on the top left corner of each image. These images were chosen to focus on the tumour and its feeding vessels. The tumour (white arrowhead) and the associated vessels appear dark, the CAM appears as grey, while the chick embryo organs are also observed as variable intensities. These images were used to generate the rendered image in Figure 7F. Data acquisition parameters are reported in the methods.

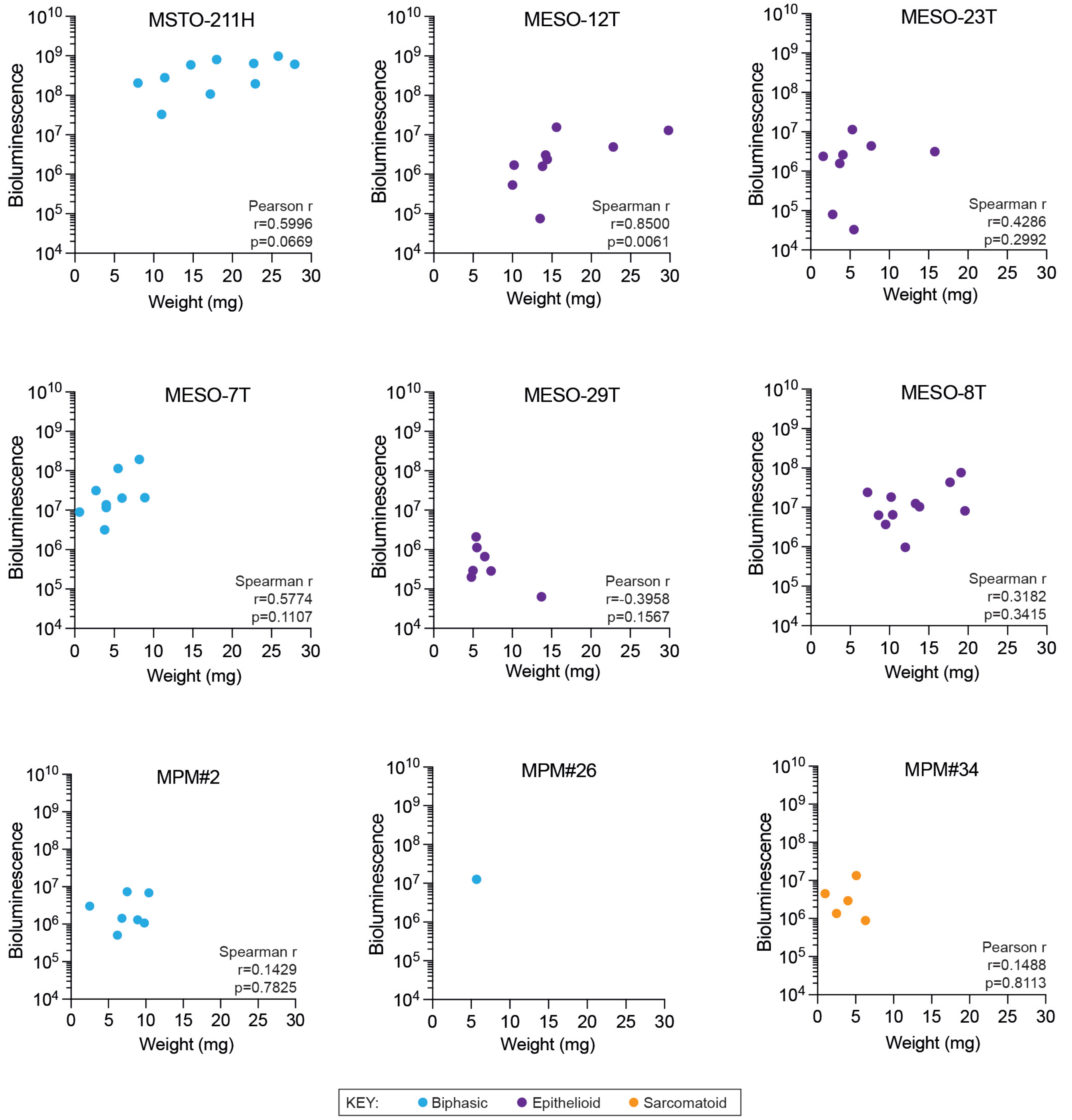

Figure S7. Tumour weight versus bioluminescence signal for individual MPM cell lines. Correlation plots of bioluminescent signal and tumour weight for individual tumours at E14 that were established from 2 million cells for each MPM cell line. Correlations are based on non-transformed data; statistical test, *r* and *p* values are indicated on each graph. These data are compiled in Figure 8C.

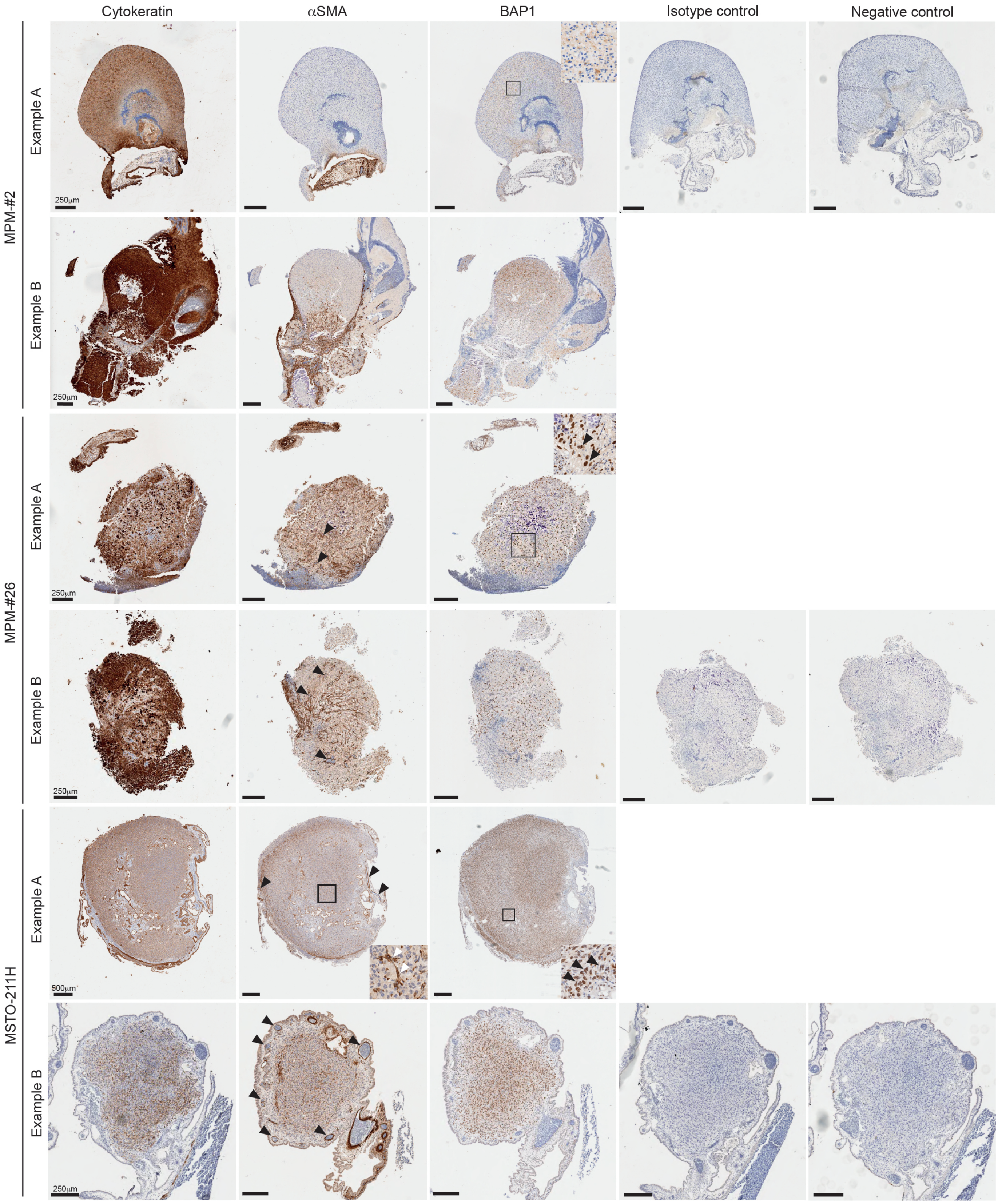

Figure S8. MPM#2, MPM#26 and MSTO-211H CAM nodule immunohistochemistry. Additional staining on adjacent sections for examples of MPM#2, MPM#26 and MSTO-211H CAM nodules in Figure 9A. Chick fibroblasts and blood vessels are stained with αSMA (arrowheads indicate small blood vessels; inset highlights infiltrating chick fibroblasts), and tumour cells with cytokeratin. MPM#2 are negative for nuclear BAP1 whilst MPM#26 and MSTO-211H are positive for nuclear BAP1 (insets, black arrowheads). Control staining was performed in parallel with an IgG negative control (Isotype control) or antibody diluent only (Negative control).

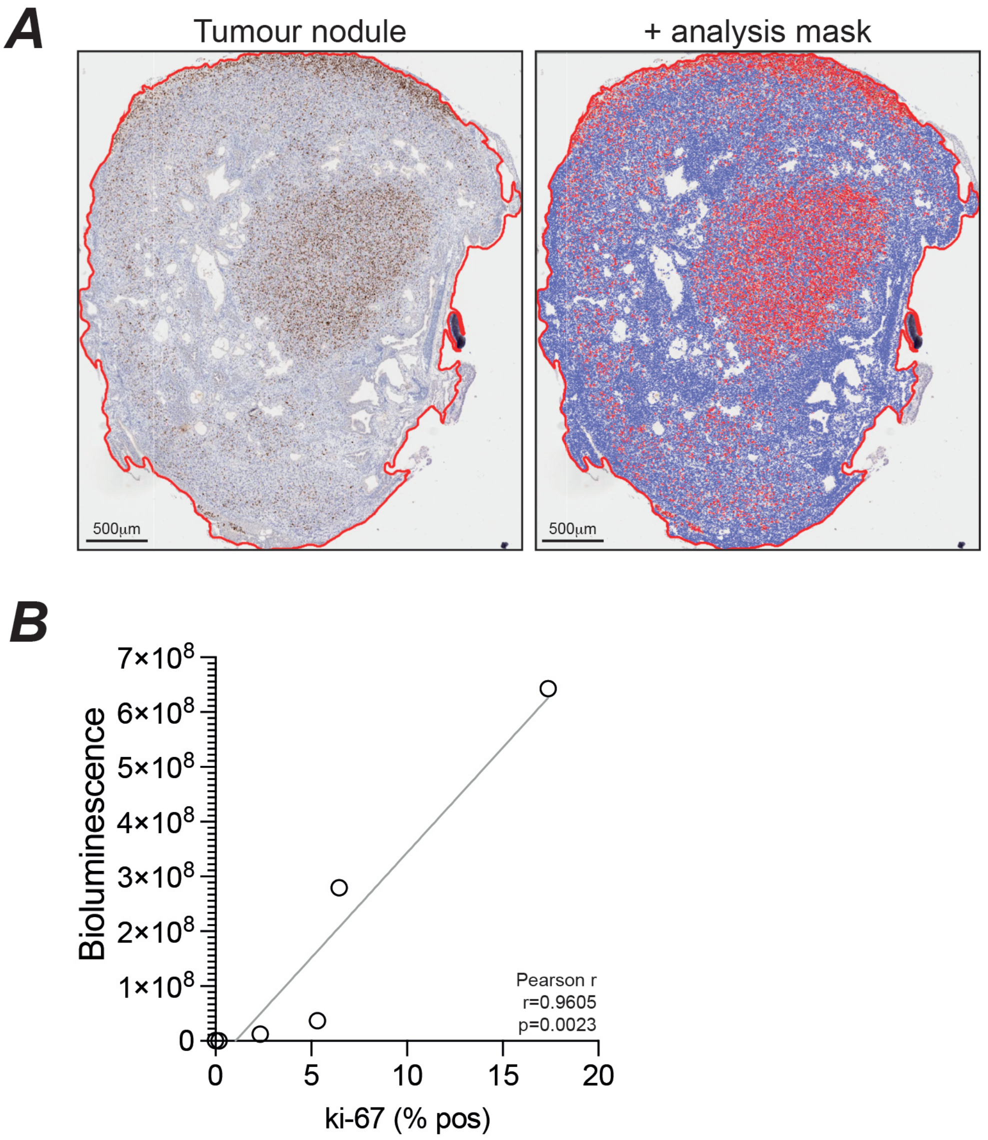

Figure S9. QuPath analsis of Ki-67 staining.

A, QuPath analysis of an MSTO-211H CAM nodule for Ki-67 staining. Tumour nodule showing Ki-67 staining (left) and with QuPath analysis mask (right); Ki-67 negative tumour or chick infiltrating cells are identified in blue and Ki-67 positive tumours cells in red (17.4%). Scale bars 500 µm. ***B,*** Correlation plot of bioluminscent signal and percentage of Ki-67-positive cells; data are from 2 nodules of each of 3 cell lines shown in Figure 9.

Table S1. Viability at E14 for eggs engrafted with MPM cells at E7. Eggs with surviving chick embryos were used to calculate engraftment rates in Figure 3B.

Embryonic day	7	14	
	n	n	% survival
● MPM#34	8	7	88
● MPM#24	10	9	90
● MSTO-211H	16	13	81
● MPM#2	14	14	100
● MESO-7T	18	12	67
● MPM#26	7	3	43
● MESO-29T	13	10	77
● MESO-12T	13	13	100
● MESO-8T	14	12	86
● MESO-23T	19	16	84

KEY: ● Biphasic ● Epithelioid ● Sarcomatoid
